# Chemical transformations of arsenic in the rhizosphere–root interface of *Pityrogramma calomelanos* and *Pteris vittata*

**DOI:** 10.1093/mtomcs/mfad047

**Published:** 2023-08-01

**Authors:** Amelia Corzo Remigio, Hugh H Harris, David J Paterson, Mansour Edraki, Antony van der Ent

**Affiliations:** Centre for Water in the Minerals Industry, Sustainable Minerals Institute, The University of Queensland, Brisbane, Australia; Department of Chemistry, The University of Adelaide, Adelaide, Australia; Australian Synchrotron (ANSTO), Melbourne, Australia; Centre for Water in the Minerals Industry, Sustainable Minerals Institute, The University of Queensland, Brisbane, Australia; Centre for Mined Land Rehabilitation, Sustainable Minerals Institute, The University of Queensland, Brisbane, Australia; Laboratory of Genetics, Wageningen University and Research, Wageningen, The Netherlands; Laboratoire Sols et Environnement, INRAE, Université de Lorraine, Nancy, France

**Keywords:** arsenic, *Pityrogramma calomelanos*, *Pteris vittata*, rhizosphere–root, speciation

## Abstract

*Pityrogramma calomelanos* and *Pteris vittata* are cosmopolitan fern species that are the strongest known arsenic (As) hyperaccumulators, with potential to be used in the remediation of arsenic-contaminated mine tailings. However, it is currently unknown what chemical processes lead to uptake of As in the roots. This information is critical to identify As-contaminated soils that can be phytoremediated, or to improve the phytoremediation process. Therefore, this study identified the *in situ* distribution of As in the root interface leading to uptake in *P. calomelanos* and *P.* vittata, using a combination of synchrotron micro-X-ray fluorescence spectroscopy and X-ray absorption near-edge structure imaging to reveal chemical transformations of arsenic in the rhizosphere–root interface of these ferns. The dominant form of As in soils was As(V), even in As(III)-dosed soils, and the major form in *P. calomelanos* roots was As(III), while it was As(V) in *P. vittata* roots. Arsenic was cycled from roots growing in As-rich soil to roots growing in control soil. This study combined novel analytical approaches to elucidate the As cycling in the rhizosphere and roots enabling insights for further application in phytotechnologies to remediated As-polluted soils.

## Graphical Abstract

**Graphical Abstract:**
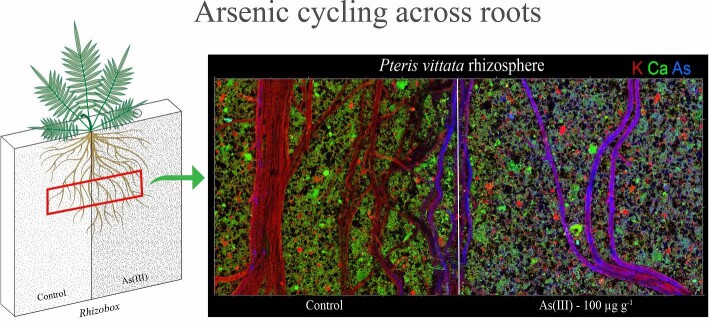
Synchrotron-based µXRF map distribution of potassium (K), calcium (Ca), and arsenic (As) in *Pteris vittata* (arsenic hyperaccumulator) showing how arsenic cycles in the roots from polluted soils to the control soil.

## Introduction

Arsenic (As) is the most toxic element on Earth,^1^ although As toxicity and bioavailability depends on the chemical form, which is highly variable.^2^ Arsenite [As(III)] is 60-fold more toxic to humans and biota compared to arsenate [As(V)].^3–6^ Arsenic is released to the environment naturally as well as through human activities. These releases pose risks for the affected ecosystems and human health, and are likely to represent a serious threat.^7^ An eco-friendly method to remove toxic metal(loid)s from local environments is *phytoextraction*, where hyperaccumulator plants are used to clean soils and water.^8–11^ In this method the aerial tissues of hyperaccumulator plants are harvested and either safely disposed, or processed for economic purposes, i.e. phytomining.^12^

Hyperaccumulating plants can accumulate metal(loid)s in their aerial tissues at concentrations up to 1000s-fold higher than normal plants.^13–15^ Typically, As concentrations in most plants are <1 µg g^−1^; however, As hyperaccumulators can reach >1000 µg g^−1^ dry wt. The arsenic hyperaccumulators are mainly fern species from the order Pteridales, genus Pteris, and accumulate extraordinarily high levels of As in their fronds.^16^ The cosmopolitan fern *Pteris vittata* can attain up to 22 600 µg As g^−1^ dry wt. in its fronds,^17^ while *Pityrogramma calomelanos* accumulates up to 8350 µg g^−1^ As dry wt.^18^ Both ferns are the strongest As hyperaccumulators; however, limited research has been devoted to *P. calomelanos* in comparison to *P. vittata*.^19^

The chemical speciation of As in fronds of *P. vittata* has been widely addressed, but studies on roots and rhizosphere are scarce.^20^ The translocation of As from roots to fronds is highly effective in *P. vittata*, and the As enrichment follows this order root < stipe < pinnae, in the latter the As concentration is higher (78–96%).^19^ Inorganic As predominates in *P. vittata*.^21^ Arsenite is dominant in the xylem sap and accounts for 93–98% of the total As, and in fronds As(III) accounts for around 80%.^22^ In the rachis and pinnules of the frond, As(III) is located in the endodermis and pericycle on the periphery of the vascular bundle system, while As(V) predominates in the vascular bundles.^19^

The main inorganic forms of As are As(III) and As(V) which are released from As-mineral weathering and represent the phytoavailable forms of As in soil solution.^23^ However, microbes contribute extensively to As inorganic and organic transformations through reduction, oxidation, methylation and demethylation reactions.^24^ The uptake of As for plants in oxidizing conditions is via As(V) through phosphate transporters, and in reducing conditions is via As(III) through silicon transporters.^25^ Another group of As analogues to As(V) are the thioarsenates (HAs^5+^S^−2^*_n_*O_4-_*_n_*^2−^; *n* = 1–4), although the uptake, and accumulation role in plants of these As species is still unclear.^26^ The oxoanion and oxoacid forms of As [As(III) and As(V)] have been well studied in the strongest As hyperaccumulator plant, *P. vittata.* In *Pteris vittata*, arsenic is cycled in the roots, As(V) enters root cells via phosphate transporters, is reduced to As(III) and some of it complexed to glutathione, and then stored in vacuoles or transported to the fronds.^27,28^ However, it is unclear how much of As(III) is complexed by glutathione in the root, and how much in the fronds.^19^ An excess of As(III) is expelled from the roots to the rhizosphere as a detoxification mechanism, where oxidation of As(III) to As(V) occurs.^29–31^ During As translocation from roots to fronds, the rhizome of *P. vittata* was found to accumulate As and regulate the preferred organ location depending on As exposure; when As is low in the substrate, the accumulation is higher in young fronds, conversely in high As environments the As accumulation is shifted to mature fronds in order to protect young tissues.^32^ In previous studies, it has been identified that the As influx in the roots of As hyperaccumulators is higher compared to non-hyperaccumulators.^32,33^ However, within As hyperaccumulators the As influx is also differentiated; e.g. a study reported that *Pteris quadriaurita* differs from *Pteris vittata* in that it does not excrete As(III) in the root efflux to control the levels of As to avoid As toxicity in the roots.^29^

A hydroponic dosing experiment showed that *P. calomelanos* in a high dose As treatment (30 mM As in the form of sodium arsenate) accumulated relatively less As in the aerial tissues (from 90% to 74%) and increased As in the roots (from 10% to 26%), compared to the lower As treatments.^34^ Arsenic probably enters *P. calomelanos* roots via the phosphate root system similar to other plants,^35^ the processes of translocation and speciation in the rhizosphere, roots, and fronds are not yet fully understood.^16^ In the fronds, the As concentration is higher in the vascular bundle, followed by the cortex, and lower in the epidermis.^36^ It is not clear how vacuoles play a role in the sequestration, internal detoxification, and chemical speciation of As in *P. calomelanos*. A hypothesis for the reason for As(V) reduction to As(III) in *P. calomelanos* is to preserve energy for metabolic function.^37^ Arsenite is toxic to plants because it interferes with sulfhydryl groups of enzymes and proteins, inhibiting cellular function^38^; while As(V), as an analogue to phosphate, interferes in ATP processes, leading to disruption of energy, and if reduced to As(III), it can trigger a similar toxicity. Once As(III) is produced, it may be complexed with sulfur (S) into less toxic forms and sequestered in vacuoles.^39^ It is still largely unclear whether As hyperaccumulator roots contribute actively to the geochemical processes in the interface of the rhizosphere, leading to As removal and uptake.^20^ Moreover, the role of root exudates in As uptake or avoidance still remains largely unknown.^40^

This study aimed to compare the *in situ* distribution of As in the root interface leading to uptake of this element in *P. calomelanos* and *P. vittata* growing in As(III) and As(V) enriched soils. Previous research has reported the speciation and translocation of As in *P. calomelanos* in spiked soil with As(V) using small freeze-dried sections of the different parts of the fern.^39^ The current study used X-ray absorption near-edge structure (XANES) imaging to map the distribution of chemical forms of As in roots of living plants growing in rhizoboxes. This approach allowed visualization of the spatial variation of As coordination environment at root level including the substrate. Synchrotron-based micro-X-ray fluorescence spectroscopy (µXRF) and laboratory µXRF were harnessed to reveal As distribution in roots and shoots of both As-hyperaccumulating ferns.

## Experimental methods

### Plant material and growing conditions

*Pityrogramma calomelanos* and *P. vittata* were propagated from original ferns growing naturally in North Queensland, following the protocol for *P. vittata*.^41^ After an incubation period (∼120 d), sporophytes were grown for 21 d and transplanted to the rhizoboxes. Infertile soil with sandy loam properties (Table 1) was collected from St Lucia Campus, University of Queensland (UQ) and used for growing the plants. The pH and electrical conductivity (EC) measurements were conducted in a 1:5 soil/water extractant.^42^ To determine the proportion of phytoavailable As an intermediate aggressive method was used,^43^ strontium nitrate extraction, 0.01 M Sr(NO_3_)_2_, at 1:4 soil to solution ratio (10 g:40 ml) with 2 h shaking before filtration and centrifugation.^44^ The measurement of Sr(NO_3_)_2_ extracts and total As concentration in soil are explained in the chemical analysis section. Soil preparation for the experiment included sieving to particle sizes under 1 mm and heat sterilization 2 h at 80°C in a drying oven.^45^ One portion of 2.1 kg of soil was spiked with As(V) to obtain the nominal concentration of 100 µg g^−1^ of As(V) by adding 874 mg of sodium arsenate dibasic heptahydrate (Na_2_HAsO_4_·7H_2_O) dissolved in 493 ml of deionized water. Another portion of 2.1 kg of soil was spiked with As(III) to obtain the nominal concentration of 100 µg g^−1^ of As(III) by adding 364 mg sodium (meta)arsenite (NaAsO_2_) dissolved in 493 ml of deionized water. For the control treatment, one portion of 2.1 kg of soil was separated without amendments. All the soil treatments reached 19% humidity, and after As dosage to soils, they were sealed and stored for 3 d, then rhizoboxes were prepared for 2 d, for further transplantation of ferns.

**Table 1.** Soil physical and chemical characteristics from the initial conditions of the control and As-enriched soil, *n* = 1 (composite bulk soil)

	C	As(III)	As(V)
Soil texture	Sandy loam	Sandy loam	Sandy loam
pH (H_2_O)^a^	5.29	5.28	5.31
Electrical conductivity (EC; µS cm^−1^)^a^	309	313	334
[As] Sr(NO_3_)_2_–extractable (µg g^−1^)	0.499	3.98	3.19
Total As (µg g^−1^)	4.85	96.8	94.6
Total P (µg g^−1^)	566	572	518
Total K (µg g^−1^)	480	602	491
Total Ca (µg g^−1^)	5440	6110	5610
Total Fe (µg g^−1^)	5760	6670	5900

^a^pH and EC were measured in 1:5 soil/water extractant (Rayment and Lyons 2011).^42^

### Rhizobox experiment

As roots grow beneath the soil surface, the observation of their foraging patterns becomes challenging. Rhizoboxes were used in this experiment to allow the observation of root growth in control and As-spiked soil. Rhizoboxes are made of transparent squared Petri dishes (containers), filled with compacted soil with the purpose of growing roots on the surface, allowing non-destructive root observation.^46^ To construct the rhizoboxes, polycarbonate square boxes were used, with upper holes for fern transplantation and watering.^47^ For this experiment, rhizoboxes without any physical barrier were divided vertically between different combinations of control soil and the As-spiked soils (left and right sides). The main goal of the rhizobox is to reach a compacted and well homogenized surface; therefore the first section was filled with the aid of a plastic barrier to immobilize the centerline of the rhizobox. The barrier was removed to fill the remaining juxtaposed section, and finally the surface was homogenized. The treatments for the rhizoboxes included control soil in the left section and As(III) or As(V)-spiked soil (100 µg As g^−1^) on the right side [C | As(III) and C | As(V)], and the combination of As(V) on left section and As(III) on the right side [As(V) | As(III)]. These three treatments were replicated three times per each fern species (*P. calomelanos* and *P. vittata*), making a total of 18 rhizoboxes. Small ferns (sporophytes allowed to grow for 21 d) were transplanted into the rhizoboxes trying to attach the roots carefully on the surface, then the rhizobox was covered with cling film, and subsequently with the Petri dish lid. Aluminum foil was used as an envelope to avoid root exposure to light. The bottoms of the rhizoboxes were lifted to 45 degrees inclination to stimulate root growth against the outer side of the rhizobox. Roots were watered daily. During the first 2 d direct light was avoided to let the ferns settle, then for 5 wk the rhizoboxes with growing ferns were kept inside a growth chamber with 12 h of 350 µmol m^2^ sec^−1^ photosynthetic photon flux density, with 20–25°C temperature and 75% humidity.

### Chemical analysis of fern tissues and soil

Once harvested, fronds were separated from the rhizoboxes and divided between young and old fronds. Roots were carefully detached from each section of the rhizobox and washed thoroughly with distilled water. Soil samples from each section of the rhizobox were also collected. All the samples were oven-dried for 72 h at 40°C. Homogenized fronds and roots subsamples were weighed **∼**100 mg (dry wt.) in 6 ml polypropylene tubes and pre-digested for 24 h with 2 ml HNO_3_ (70%). The digestion was conducted on a heated block (Thermo Scientific Digital Dry Bath) in two steps: 1 h at 70°C, followed by 1 h at 125°C. Ultrapure water (Millipore 18.2 MΩ cm at 25°C) was added to the digested samples to make 10 ml for further analysis. Soil subsamples of 100 mg (dry wt.) were poured into quartz tubes to add reverse aqua regia: 5 ml of HNO_3_ (70%) and 2 ml of HCl (37%). The digestion was performed using a ColdBlock SB15S Digester during four rounds of 240 s each (totaling 16 min). The ColdBlock system uses focused infrared radiation to speed the sample breakdown and a cooling system to regulate the temperature.^48^ After digestion of fern tissues and soil samples, along with the Sr(NO_3_)_2_ extracts, samples were analysed for macroelements (Al, Ca, Na, Mg, K, P) and trace elements (As, Cu, Fe, Mn, Zn) using the Thermo Scientific iCAP 7400 inductively coupled plasma atomic emission spectroscopy (ICP–AES) instrument. Either radial or axial mode was operated considering the approximate element concentration in the analyte. Matrix-based interferences were compensated using yttrium in-line internal addition standardization. Quality controls included blanks and certified reference material (Sigma-Aldrich Periodic table mix 1 for ICP TraceCERT®, 33 elements, 10 mg L^−1^ in HNO_3_) and standard reference material for plants and soil (NIST Apple 1515, and NIST Estuarine Sediment 1616).

### Laboratory-based X-ray fluorescence imaging

The µXRF facility at UQ is a customized IXRF ATLAS X system which has a 50-Watt microfocus Mo-tube that produces 17.4 keV X-rays with a 2.2 × 10^8^ ph s^−1^ flux focussed to a 25 µm spot. The system is equipped with two silicon drift detectors (150 mm^2^), and the elements that can be measured under air are from aluminum (Al) to selenium (Se). The measurements were carried out using 40 kV accelerating voltage on the tube and a 100 ms per-pixel dwell time at room temperature. Fresh/live samples were mounted on a stage and covered with 4 µm Ultralene thin film to avoid evaporation in plant specimens, and analysed within 10 min of excision. In this study, radiation-induced damage was negligible, as the source yielded a 2.2 × 10^8^ photons s^−1^ flux in a 25 µm beam spot, and maximum dwell time recorded was 100 ms, resulting in only 6.6 Gy deposited radiation, considerably less from the threshold identified for hydrated plant organs.^49^ The Iridium (IXRF systems) instrument control package was utilized to acquire the XRF spectra in mapping mode.

### Synchrotron-based X-ray fluorescence and X-ray absorption near-edge structure imaging

Synchrotron radiation allows *in situ* spectroscopic techniques; XFM unravels the elemental spatial distribution while XANES imaging allows the spatial distribution of different chemical forms of an element to be determined.^50^ The XFM beamline of the Australian Synchrotron employs an in-vacuum undulator to produce a brilliant X-ray beam with a focus down to 1 µm. A double crystal monochromator Si(111) and Kirkpatrick–Baez mirrors produce a monochromatic incident beam focused onto the sample.^51,52^ The beamline is equipped with a Maia XRF detection system which enables short pixel times (sub-millisecond) and large pixel counts (megapixels) for high-definition imaging.^53^ The XRF mapping used an incident energy of 15.8 keV. The spatial variation in As chemical speciation was assessed with XANES maps of selected areas of live plants and hydrated soil. The fluorescence 2D XANES imaging comprised “stacks” of µXRF images, collected by scanning the sample area several times at successive incident energies (a series of 112 energies across the As-edge was used with a 10 ms/energy/spatial point dwell time). Consequently, the selected area of the sample was raster-scanned over a 2.380 × 1.295 mm window with a 5 µm step size, yielding a µ-XANES image stack of 477 × 260-pixel images. The specimen was scanned over the As K edge (∼11 867 eV) at 112 energies from 11 800 to 12 100 eV, using 5 eV steps across the pre-edge region (11 800–11 855 eV), 0.5 eV steps across the XANES region (11 855–11 900 eV), and 20 eV across the post-edge region (11 900–12 100 eV). Disruptions and access challenges resulting from restrictions in response to the COVID-19 pandemic in Australia meant that the quality of energy calibration achieved for this data collection was not optimal. Energy calibration was achieved by calibrating the double crystal monochromator to the first peak of the first derivative of a spectrum of an in vacuum thin copper foil at 8980.3 eV. A further calibration energy correction of 0.80 eV was then applied assuming that the highest energy peak observed in the spectra extracted from the soil/plant XANES images corresponded to the white line of the pH 9 As(V) standard (11 875.5 eV) used in the XANES fitting analyses. There is likely a greater level of uncertainty in the XANES fitting results described in the following text than might otherwise be expected from such an analysis. The impact of this uncertainty on the interpretation of the data analysis for these experiments is addressed in the discussion in the following text.

### Data analysis

The data from the XFM were processed using the GeoPIXE software package as described earlier.^54,55^ The matrix used for XRF spectra modelling was a soil with the empirical ratio metric formula of C_2_O_20_Si_5_Mg_0.18_K_0.3_Ca_0.69_Fe_2.43_ (based on the bulk chemical composition of the soil) with a density of 1.6 g cm^3^ and a thickness of 20 mm (e.g. thickness of the rhizobox). The XANES mapping produced a 3D image stack of spatial dimensions (*x, y*) and an X-ray incident energy-resolved dimension E (energy). Pixels with similar spectra were classified together by *k*-means clustering, as a result three different clusters were identified and associated with the root, soil, and image background. The data from the UQ µXRF were processed as described earlier^56,57^ and visualized using ImageJ's “Fire” lookup table.

XANES spectra extracted from spatial regions from roots and soil were fit to linear combinations of model compound spectra reported previously^28^ (see Supplementary Fig. S1), using the program DATFIT which is part of the EXAFSPAK suite of programs (G. N. George, Stanford Synchrotron Radiation Laboratory). A spectrum of a sample of solid sodium dithioarsenate collected at the Australian Synchrotron XAS beamline (also calibrated to the As(V) white line peak energy, provided by Jason Kirby, CSIRO) was additionally included in the linear combination fitting. Although this model was not well characterized, the spectrum collected is consistent in terms of peak height and energy position with previously reported examples in the literature.^58^

### Statistical analyses

The As concentration in different organs of *P. calomelanos* and *P. vittata* were compared using statistical analyses. The statistical analysis of variance, Shapiro–Wilk test of normality (*p* > 0.05) was first performed to check the assumption of normality, and then the homogeneity of variance assumption was evaluated with the Levene's *F*-test (*p* > 0.05). The significance of differences in As concentration in fronds (young and old) resulting from the independent treatments in each fern species were assessed using three-way ANOVA. Differences in As concentration in roots in the As-spiked and control zones across all treatments and both fern species were assessed through three-way ANOVA. A Student's *t*-test was performed to evaluate As accumulation in roots within left and right root sections for each treatment and fern species. R software version 4.0.2 was used for the statistical analyses and plots, with a significance level of *p* < 0.05.

## Results

### Arsenic distribution in the rhizoboxes

Potential As phytoavailability (operationally defined as the Sr(NO_3_)_2_-extractable fraction) and total As concentration in control soil and As-spiked soil are summarized in Table 2. Across the C | As(III) and C | As(V) treatments in the rhizoboxes, phytoavailable As in control soil was found in a range of 0.151–0.872 µg As g^−1^, while total As was 1.27–2.79 µg As g^−1^. In treatments with As-spiked soil supplied either as As(III) or As(V), the phytoavailable As was slightly higher in the As(III) treatment with a range of 3.55–4.21 µg As g^−1^, while in the As(V) treatment it was 2.61–4.06 µg As g^−1^. The total As concentration in the As(III)-spiked soil was slightly lower compared to the As(V)-spiked soil, with a range of 83–88 µg As g^−1^, while the As range in As(V)-spiked soil was 85.1–104 µg As g^−1^. Comparing the rate of As depletion between *P. calomelanos* and *P. vittata*, the latter tends to remove more As from the control and As-spiked treatment, given that the results show that the total As concentration is lower in the soil from *P. vittata* rhizoboxes. For example, in the C | As(V) treatment, the As concentration in the control soil was 1.83 µg g^−1^ for *P. vittata* and 2.79 µg g^−1^ for *P. calomelanos*, and in the As-spiked soil was 85.1 µg g^−1^ and 92.8 µg g^−1^, respectively.

**Table 2.** Soil parameters in the rhizoboxes across the As treatments, measured after *Pityrogramma calomelanos* and *Pteris vittata* harvest (dry wt.)

	Soil treatments					
	C ǀ As(III) (100 µg g^−1^)		C ǀ As(V) (100 µg g^−1^)		As(V) ǀ As(III) (100 µg g^−1^)	
*Pityrogramma calomelanos*	C	As(III)	C	As(V)	As(V)	As(III)
[As] Sr(NO_3_)_2_–extractable (µg g^−1^)	0.872 ± 42.2	3.89 ± 11.4	0.72 ± 32.4	2.59 ± 14.8	4.06 ± 14.9	3.64 ± 27.8
[As] total concentration in soil (µg g^−1^)	2.2 ± 90.1	83 ± 6.07	2.79 ± 90.7	92.8 ± 3.82	104 ± 14.2	86.2 ± 12.2
pH	5.95 ± 2.91	5.85 ± 1.24	6.19 ± 2.52	6.3 ± 2.17	6.23 ± 1.96	6.12 ± 2.38
EC (µS cm^−1^)	180 ± 18.5	197 ± 18.5	122 ± 17	123 ± 30.8	161 ± 18.4	165 ± 29.5
***Pteris vittata***	**C**	**As(III)**	*C	*As(V)	*As(V)	*As(III)
[As] Sr(NO_3_)_2_–extractable (µg g^−1^)	0.151 ± 92.2	3.55 ± 39.3	0.408, 0.182	2.39, 2.84	4.3, 3.39	4.73, 3.7
[As] total concentration in soil (µg g^−1^)	1.27 ± 83	88 ± 4.66	0.788, 2.87	86.09, 84.02	94.1, 98.9	80.1, 78.7
pH	6.33 ± 0.885	6.26 ± 1.38	6.47, 6.47	6.46, 6.41	5.89, 6.14	6.06, 6.44
EC (µS cm^−1^)	125 ± 28.2	135 ± 30.2	99.8, 102.7	107.8, 97.7	265, 108	206, 114

Means ± relative standard deviation (%RSD), *n* = 3, *n = 2 ferns died during transport from Melbourne to Brisbane. Rhizobox treatments: (left/right sides); C: control, As(III): arsenite, As(V): arsenate, 0 and 100 µg g^−1^ are the given initial As concentrations in control and As-enriched soils.

### Arsenic concentrations in *P. calomelanos* and *P. vittata*

**Fig. 1:**
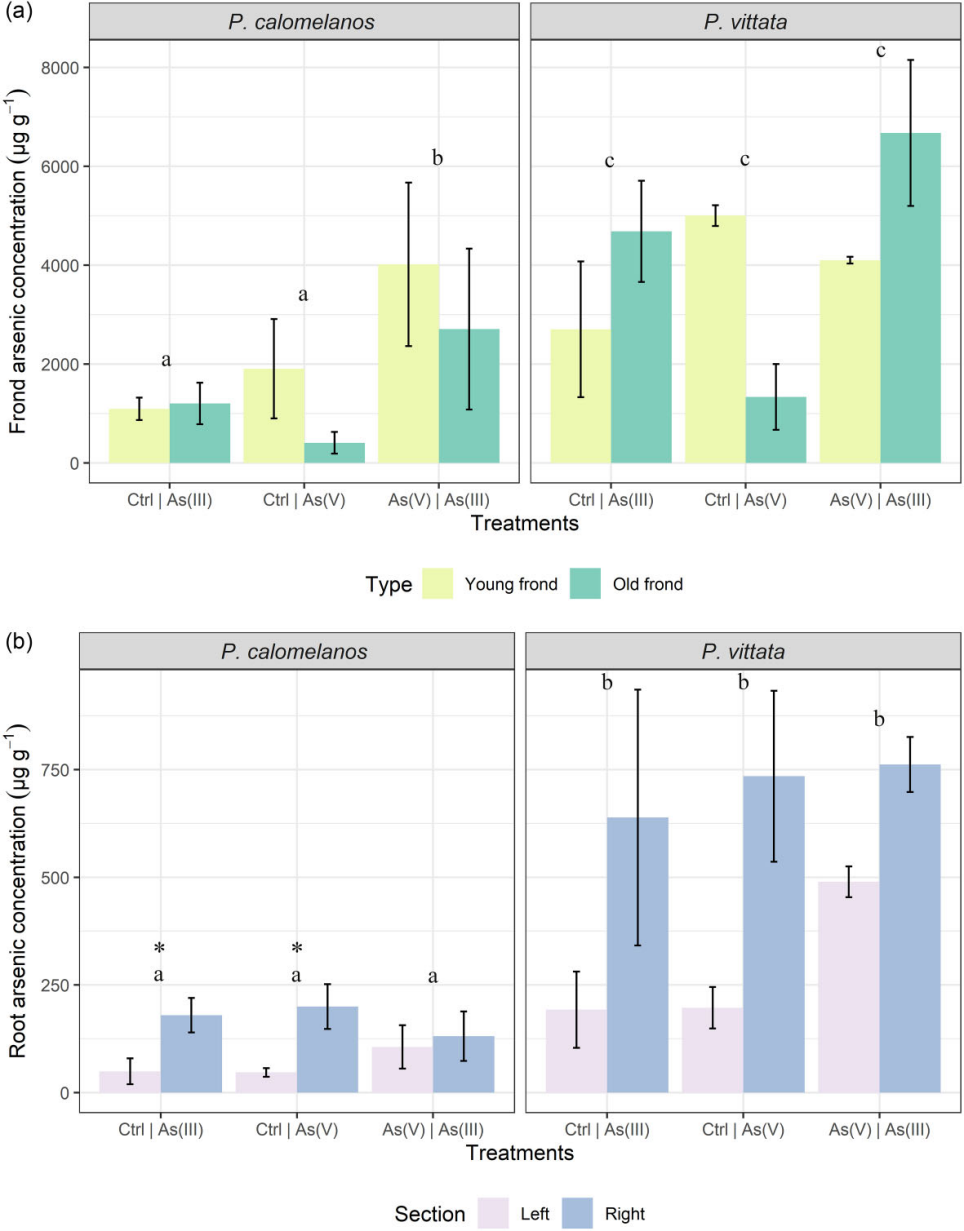
Arsenic concentration (µg g^−1^) in fronds and roots of *P. calomelanos* and *P. vittata* grown in mixed treatments with control and As-spiked soil: (a) As concentration in young and old fronds, means ± standard deviation (*n* = 3) are assessed using three-way ANOVA across the treatments, type of fronds, and fern species, followed by Bonferroni *post hoc* test (*p* < 0.05); (b) As accumulation in left- and right-hand roots, means ± standard deviation (*n* = 3) are assessed using three-way ANOVA across the treatments, root section, and fern species, followed by Bonferroni *post hoc* test (*p* < 0.05). Different letters show statistical difference in the grouped bars. Student's *t-*test was performed to compare left- and right-hand As concentration in roots within each treatment (considering the same replicates); * denotes a statistical difference of *p* < 0.05.

Arsenic concentration in fronds and roots are shown in Table 3. Frond As uptake in both fern species subjected to the three different treatments [C | As(III), C | As(V), As(V) | As(III)] were statistically significant different [*F*(2, 19) = 4.710, *p *= 0.022]. The As uptake was also statistically significantly different depending on the fern species [*F*(1, 19) = 33.637, *p* = 0.005], and the treatment [*F*(2, 19) = 12.581, *p* = 0.0003]. In Fig. 1a, the As accumulation was higher in the mixed treatment As(V) | As(III), and *P. vittata* accumulation was higher than *P. calomelanos*, with 6670 µg As g^−1^ on average in the old fronds, compared to 4020 µg As g^−1^ in the young fronds of *P. calomelanos*. The As concentration in the roots was statistically different depending on the fern species [*F*(1, 20) = 84.906, *p* < 0.001], and depending on the position of the roots (e.g. left or right side) [*F*(1, 20) = 36.081, *p* < 0.001]. To evaluate differences in root As concentrations in each treatment, a Student's *t-*test was performed. In *P. calomelanos*, the independent *t-*test in C | As(III) and C | As(V) yielded a significant difference (*p* < 0.05), while the treatment As(V) | As(III) did not report a statistically significant difference (*p* *=* 0.602). In *P. vittata*, the independent *t-*test did not show any differences between the control and the spiked As soil (*p* < 0.05). In Fig. 1b, the roots exposed to As-spiked soil accumulated more As in both fern species, although *P. vittata* attained also higher As concentration in the control soil compared to *P. calomelanos.* For example, in the C | As(III) treatment, the As concentration in roots in the control soil was 193 µg As g^−1^ in *P. vittata*, but only 49.6 µg As g^−1^ in *P. calomelanos*. In general *P. vittata* accumulated more As in its roots compared to *P. calomelanos*. In the As(V) | As(III) treatment, *P. vittata* concentrated 489 µg As g^−1^ in the left side As(V), and 762 µg As g^−1^ in the As(III) right side, compared to *P. calomelanos* 106 µg As g^−1^ in the As(V) left side, and 131 µg As g^−1^ in the As(III) right side.

**Table 3.** As-enriched soil effects on As accumulation by *Pityrogramma calomelanos* and *Pteris vittata* grown in rhizoboxes

	*Pityrogramma calomelanos*			*Pteris vittata*		
	C ǀ As(III) (100 µg g^−1^)	C ǀ As(V) (100 µg g^−1^)	As(V) ǀ As(III) (100 µg g^−1^)	C ǀ As(III) (100 µg g^−1^)	C ǀ As(V) (100 µg g^−1^) *	As(V) ǀ As(III) (100 µg g^−1^) *
[As] in young fronds (µg g^−1^)	1100 ± 20.5	1900 ± 52.8	4020 ± 41.2	2700 ± 50.7	5150, 4860	4050, 4150
[As] in old fronds (µg g^−1^)	1210 ± 34.8	408 ± 53.9	2710 ± 60.1	4680 ± 21.8	865, 1800	7720, 5630
[As] in left roots (µg g^−1^)	49.6 ± 86.1	47 ± 21.3	106 ± 47.4	193 ± 45.8	231, 163	515, 464
[As] in right roots (µg g^−1^)	180 ± 22.2	200 ± 26	131 ± 43.9	639 ± 46.5	875, 594	717, 807
Young fronds mass (mg)	55 ± 31.3	36.6 ± 44.9	46 ± 22.7	29.2 ± 37	7, 9.7	33.8, 29.5
Old fronds mass (mg)	121 ± 22.2	39 ± 27.3	107 ± 51.7	29.5 ± 44.8	27.6, 19.2	37.1, 42.3
Total fronds mass (mg)	176 ± 11.9	75.6 ± 31.7	153 ± 37.2	58.8 ± 10.1	34.6, 28.9	70.9, 71.8
Left root mass (mg)	107 ± 24.5	42.6 ± 81.7	46.7 ± 31.6	24.8 ± 39.2	8.7, 7.2	16.7, 14.4
Right root mass (mg)	112 ± 6.78	21 ± 97	72.5 ± 45.6	28.9 ± 40.7	5, 5.7	35.7, 8.8
Total root mass (mg)	219 ± 15.1	63.6 ± 85.7	119 ± 39.2	53.7 ± 39.2	13.7, 12.9	52.4, 23.2
Root:frond mass ratio	1.24 ± 10.6	0.757 ± 54.3	0.807 ± 35.7	0.927 ± 44.9	0.4, 0.446	0.74, 0.323

Means ± relative standard deviation (%RSD), *n* = 3, *n = 2 ferns died during transport from Melbourne to Brisbane. Rhizobox treatments: (left/right sides); C: control, As(III): arsenite, As(V): arsenate, 0 and 100 µg g^−1^ are the given initial As concentrations in control and As-enriched soils. The elemental analysis was performed in dry weight samples.

### Tissue-level distribution of As in *P. calomelanos* and *P. vittata* fronds

The laboratory µXRF elemental map shows the distribution of As, Ca, and K in the young and old fronds of both fern species (Supplementary Fig. S2). In agreement with the analysis of total elemental concentrations, the older fronds accumulated higher As than the young fronds. In the magnified elemental map distribution of *P. vittata* (Supplementary Fig. S2), the As was enriched in the rachis (mid-rib) along the blade, costa (midrib of the pinna), and somewhat depleted in the vascular bundles, although it was higher in the terminal veins. The K distribution was similar to that for As, albeit higher in the base of the blade, while Ca was different as it was higher in the rachis and costa, but depleted in the pinna, and somewhat enriched on the margins. In the younger blade of *P. vittata*, the Ca was higher across all the blade, mirroring the chlorotic damaged tissues. The As accumulation in *P. calomelanos* was predominantly noted in the rachis, and even more abundant in the pinna base and costa, but absent from the margins, which was distinct from the As distribution observed in *P. vittata*. The distribution of K in *P. calomelanos* followed the same pattern as As, although more abundant, while Ca was evenly distributed across the blade, with a clear enrichment in the damaged tissues (see Supplementary Fig. S2).

### Synchrotron XFM analysis of *P. calomelanos* and *P. vittata* roots

**Fig. 2:**
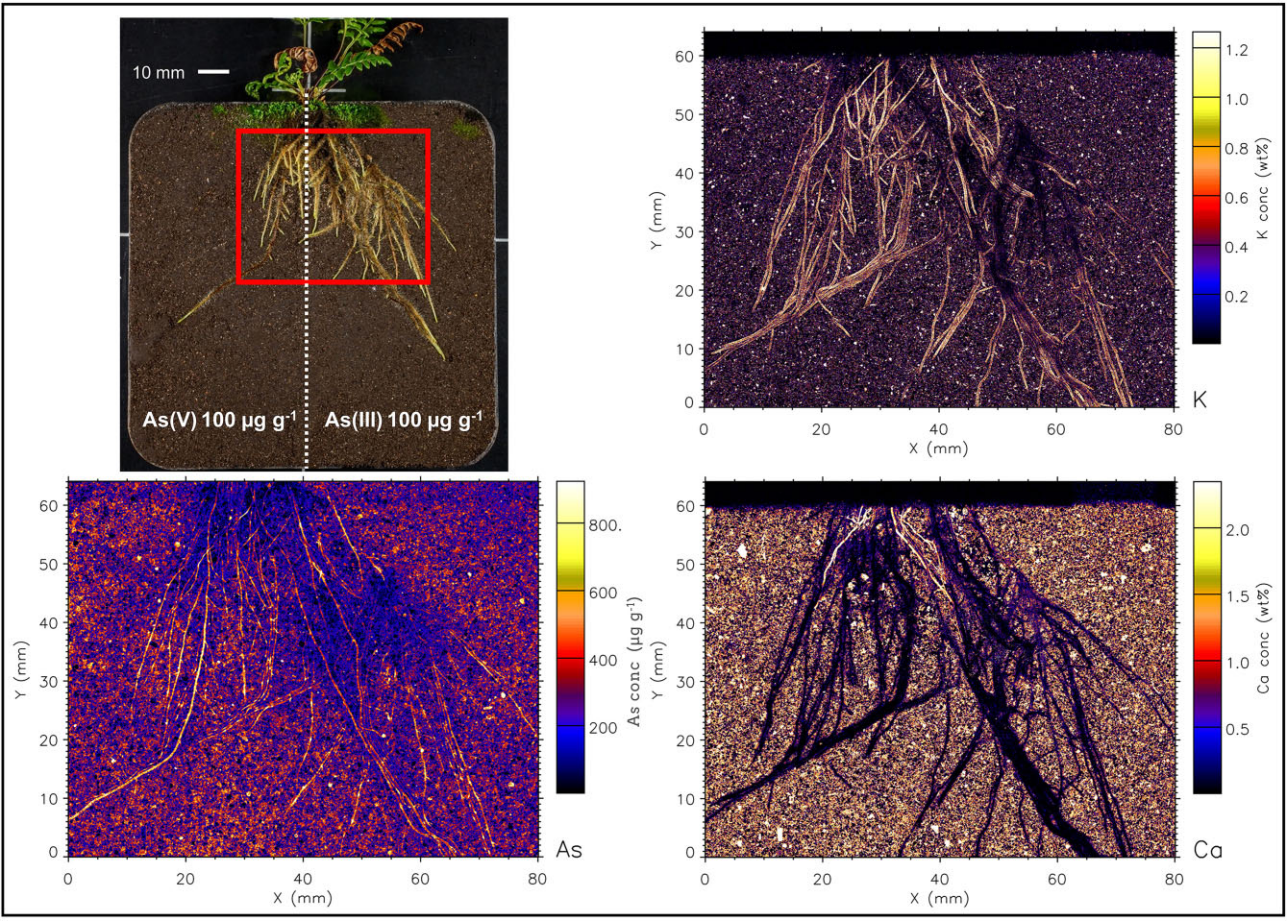
Synchrotron µXRF elemental maps displaying K, As, and Ca distribution in a representative area of the left and right sides of the rhizobox with *Pityrogramma calomelanos* fern, grown in As(V) | As(III) treatment, third replicate.

**Fig. 3:**
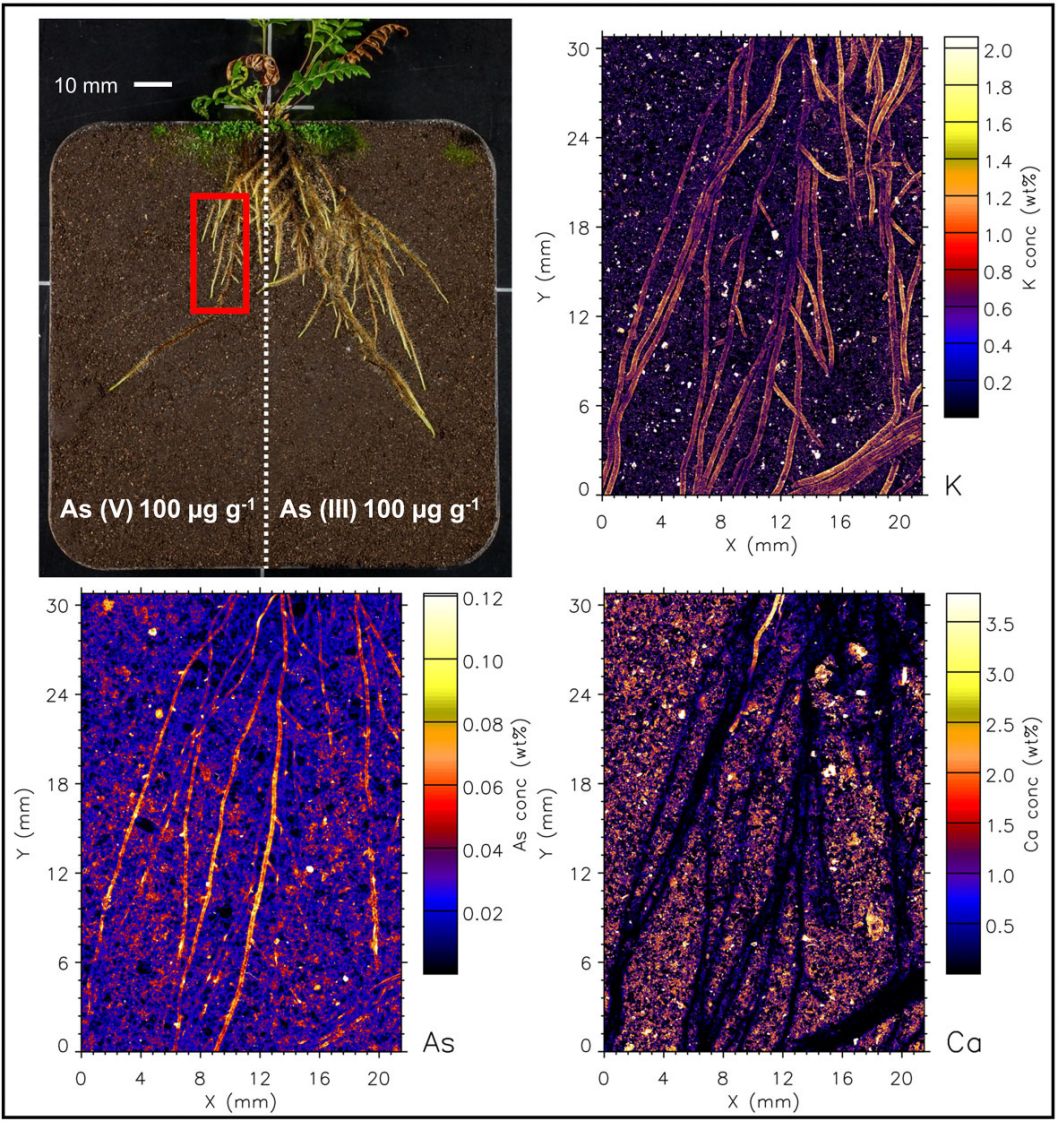
Synchrotron µXRF elemental maps displaying K, As, and Ca distribution in the left side of the rhizobox [As(V)-spiked soil] with *Pityrogramma calomelanos*, grown in As(V) | As(III) treatment.

**Fig. 4:**
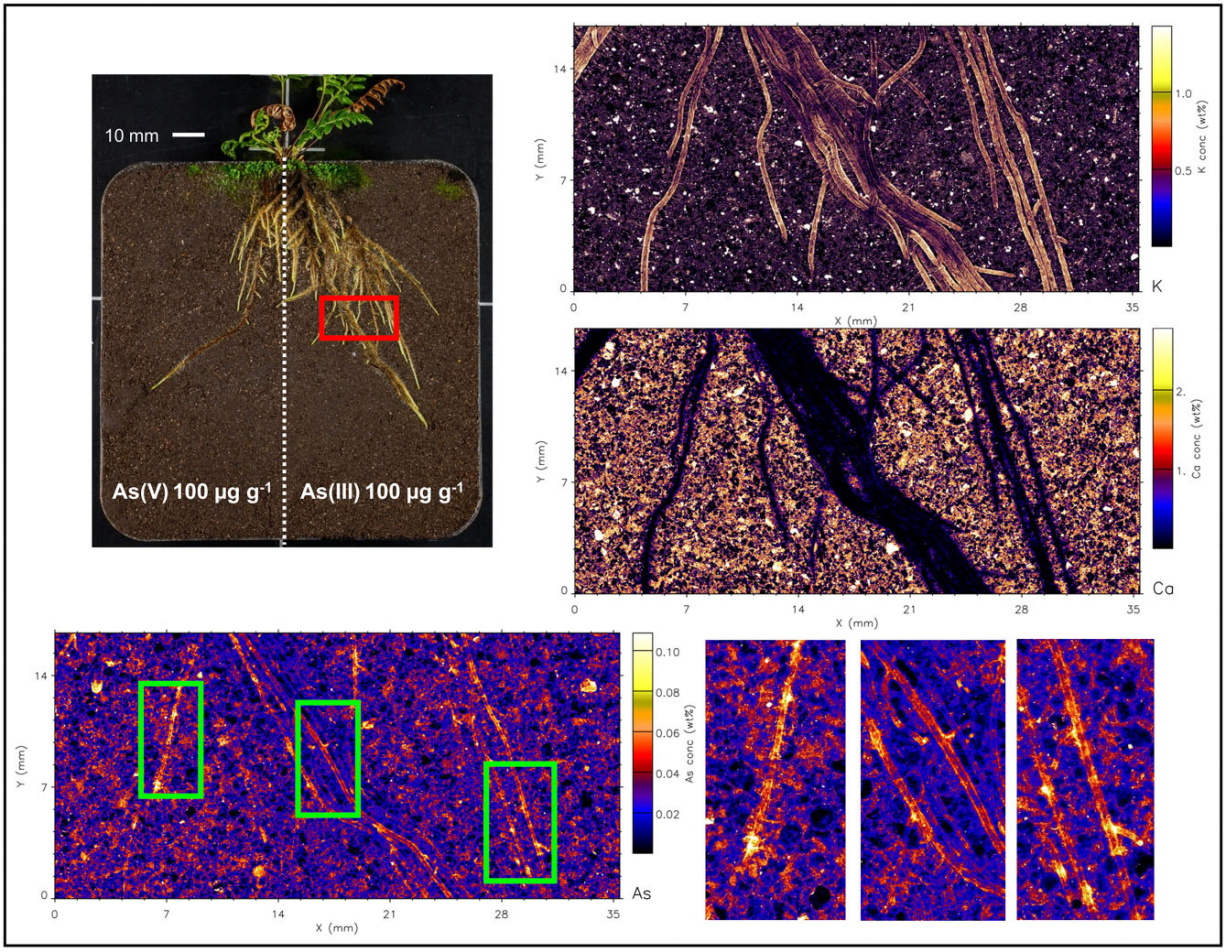
Synchrotron µXRF elemental maps displaying K, As, and Ca distribution in the right side of the rhizobox (As(III)-spiked soil) with *Pityrogramma calomelanos*, grown in As(V) | As(III) treatment.

**Fig. 5:**
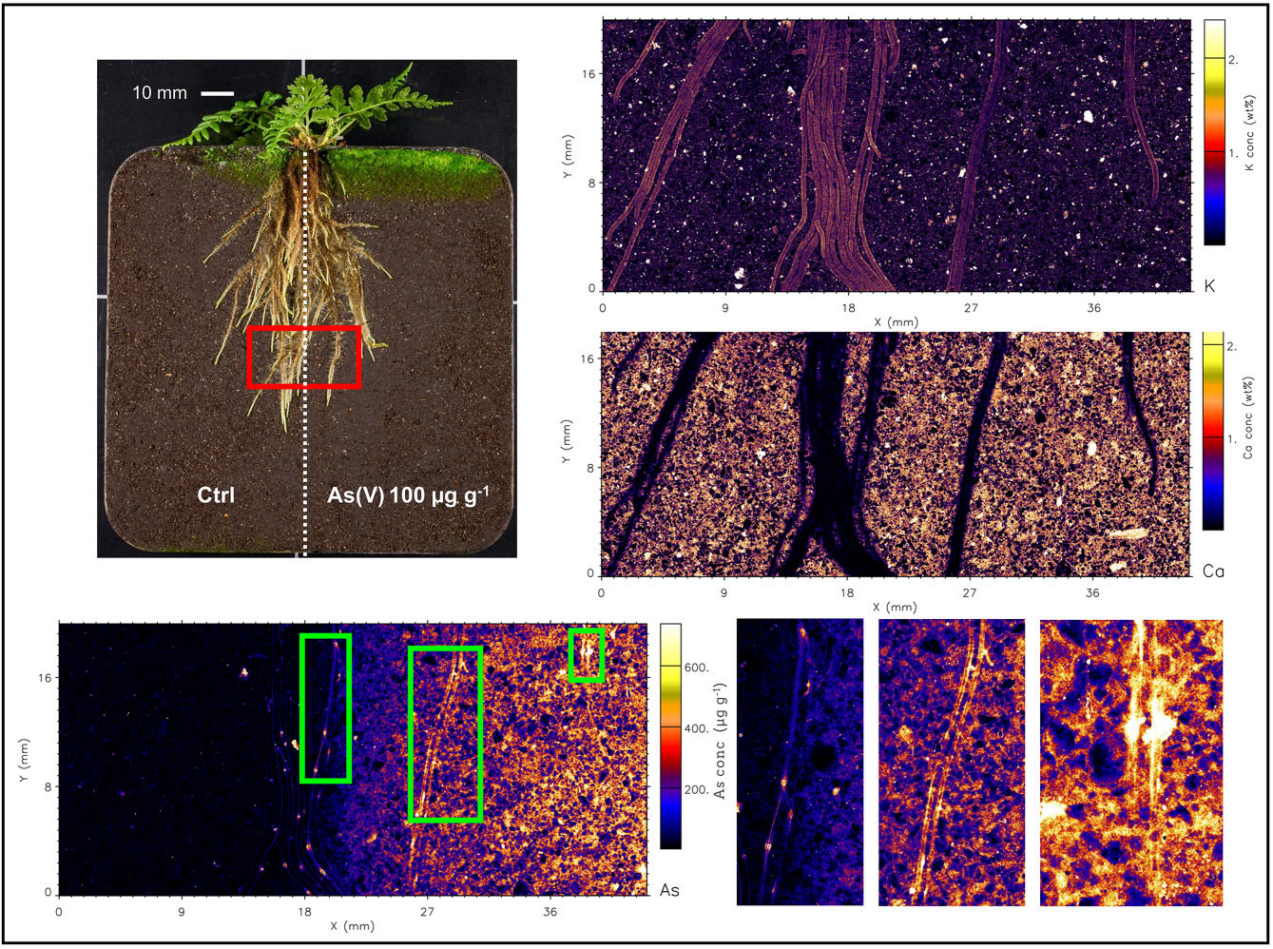
Synchrotron µXRF elemental maps displaying K, As, and Ca distribution in a small representative area of the left and right sides of the rhizobox with *Pityrogramma calomelanos* fern, grown in C | As(V) treatment.

**Fig. 6:**
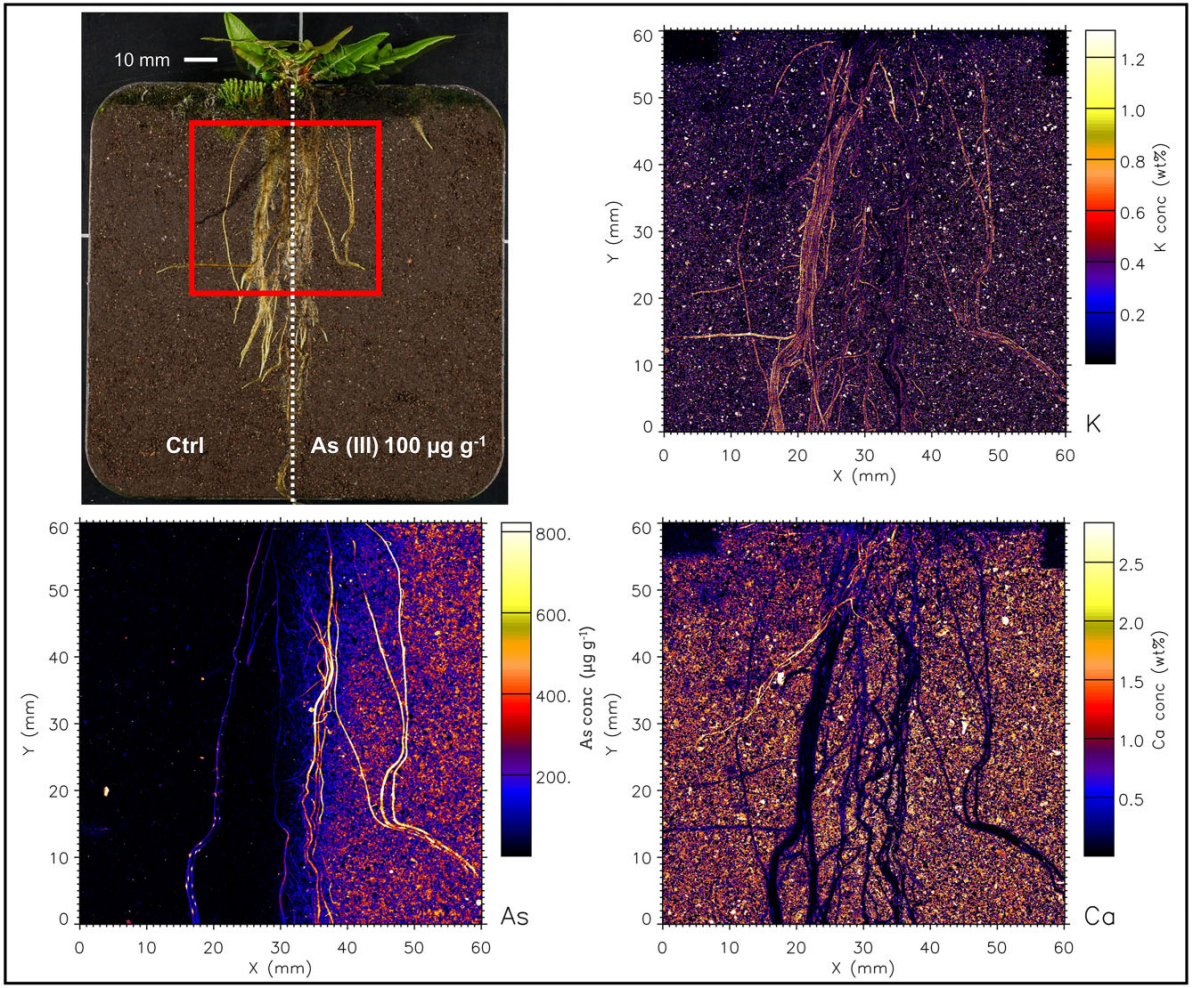
Synchrotron µXRF elemental maps displaying K, As, and Ca distribution in a representative area of the left and right sides of the rhizobox with *Pteris vittata* fern, grown in C | As(III) treatment.

**Fig. 7:**
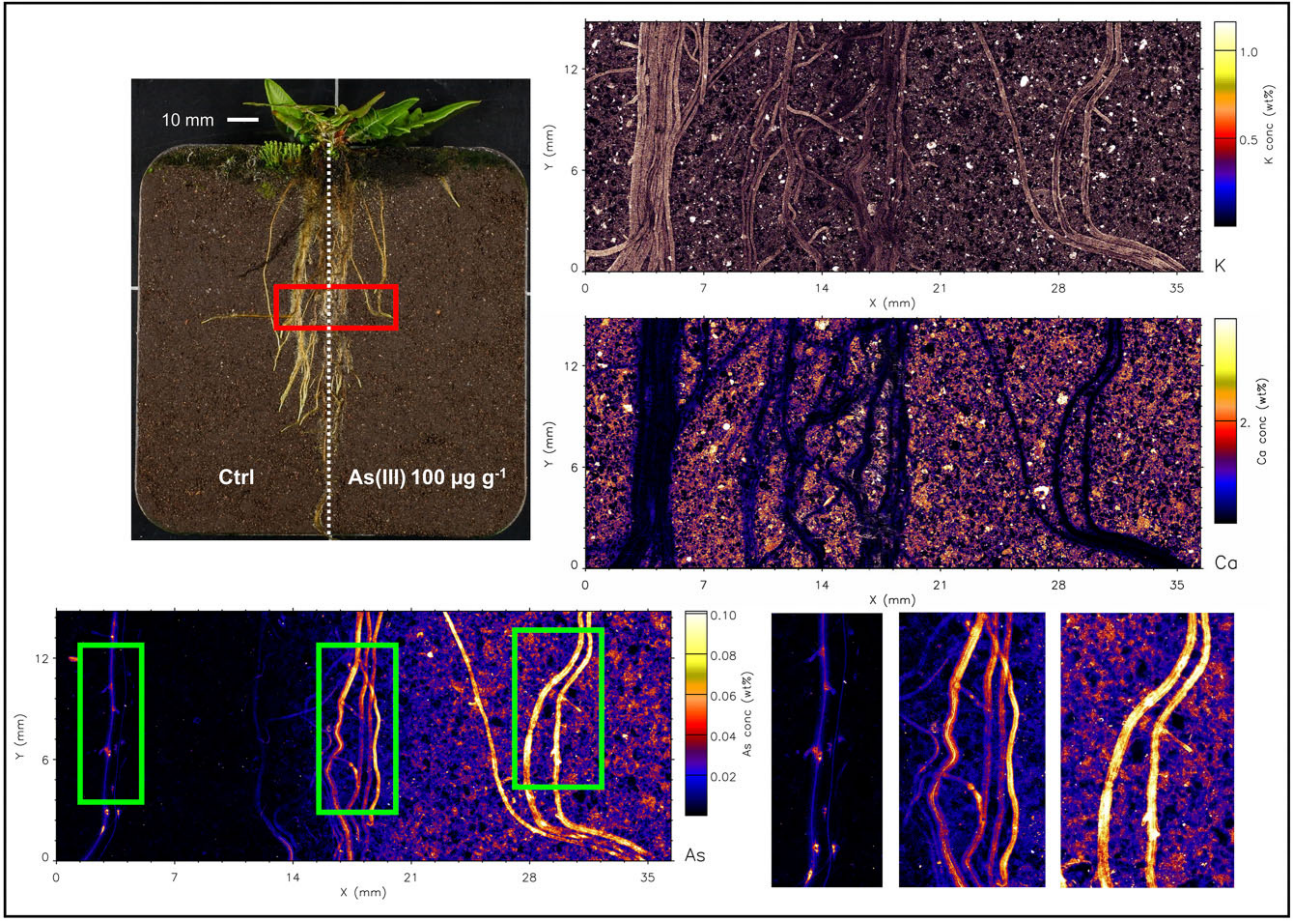
Synchrotron µXRF elemental maps displaying K, As, and Ca distribution in a small representative area of the left and right sides of the rhizobox with *Pteris vittata* fern, grown in C | As(III) treatment.

Even though As was supplied to soil in water soluble form, the µXRF maps (and elemental analysis) show that As is not mobile and has not migrated from the treated side to the control side. The elemental maps of roots are shown in Figs. 2–7, and Supplementary Figs. S3–5. In both fern species, K concentration was higher in roots than the soil background, while Ca concentration was in general lower in roots than in soil. The As in the roots of both species in the As-enriched soil was present in high concentrations, and in the control soil section the As concentration in roots was observed cycling—we hypothesize from roots to xylem, shoot, phloem and roots^59^—although in lesser amounts (see also Supplementary Figs. S3 and S5). Despite the similarities of the As distribution across both species, there were subtle differences; e.g. As concentration in *P. vittata* roots tended to be higher compared to *P. calomelanos.* In the As(V) | As(III) treatment, the As enrichment in *P. calomelanos* roots was more prominent in the As(V)-enriched soil (see Figs. 2 and 3), while slightly lower in the As(III)-enriched (see Fig. 4). In the same mixed treatment [As(V) | As(III)], the As concentration in *P. vittata* roots in both As-enriched sections was evenly distributed (see Supplementary Fig. S4). In the C | As(V) treatment, *P. calomelanos* clearly cycled As throughout the roots growing in the control soil section, although As in roots was slightly lower (similar in *P. vittata—*see Supplementary Fig. S5) but presented some nodes with high concentrations (see Fig. 5). As-enriched nodes in roots growing in the control soil were also evident in *P. vittata* (C | As(III) treatment), although the As concentration in roots subjected to the As(III)-enriched section was considerably higher in comparison to those in *P. calomelanos* (see Figs. 6 and 7).

### XANES analysis of *P. calomelanos* and *P. vittata* roots

**Fig. 8:**
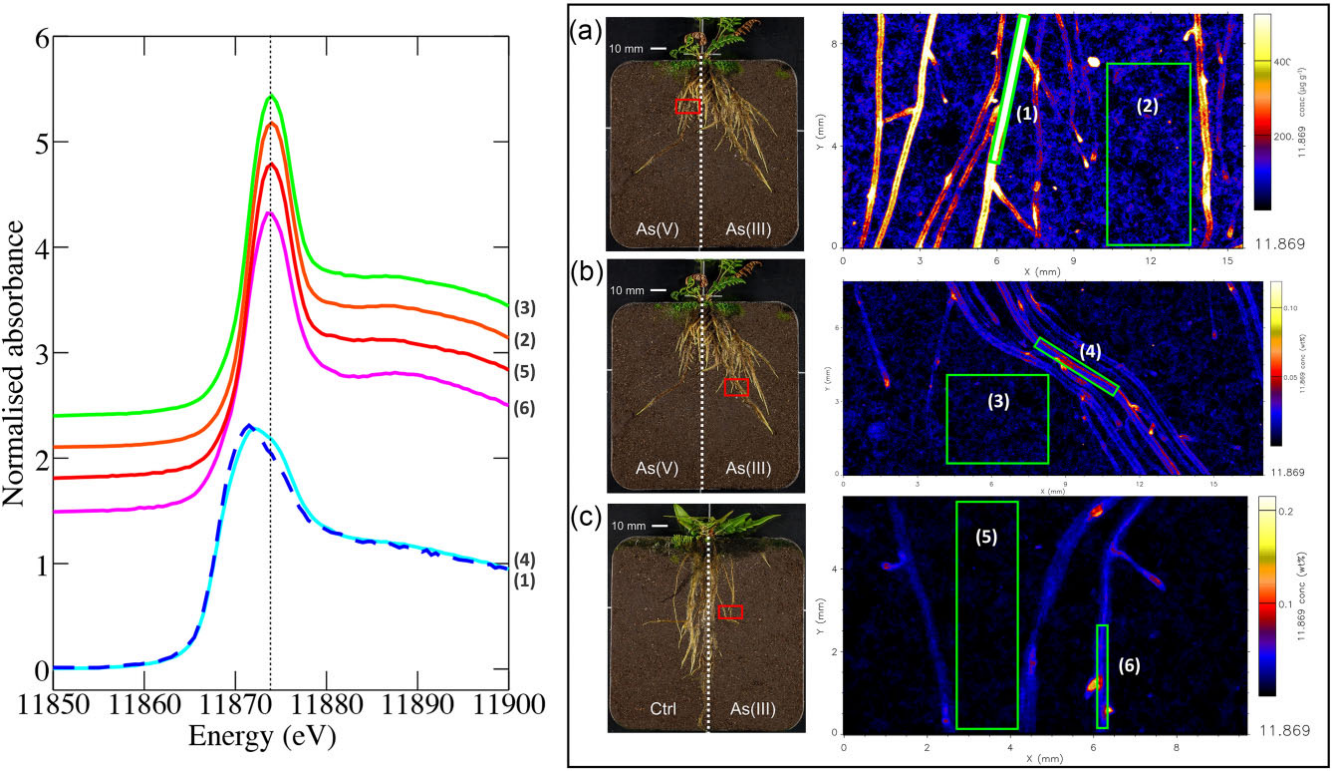
As K-edge X-ray absorption near edge spectra from six samples: (a) *P. calomelanos*—region [As(V)-spiked soil]: root area spectra (1)—(aqua), and soil area spectra (2)—(orange); (b) *P. calomelanos—*region [As(III)-spiked soil]: soil area spectra (3)—(green), and root area spectra (4)—(blue); (c) *P. vittata—*region [As(III)-spiked soil]: soil area spectra (5)—(red), and root area spectra (6)— (purple). Dashed vertical line is 11875.5 eV.

Root and soil regions selected for the XANES fitting analyses and the extracted spectra are shown in Fig. 8, and the results of the linear combinations fitting of model compound spectra to the extracted spectra are summarized in Table 4. The As K-edge XANES spectra extracted from the soil-only regions from all examined treatments were visually identical to each other and very similar to the spectrum from the root of *P. vittata* grown in As(III) amended soil. XANES fitting of these four spectra showed As(V) to be the dominant chemical form of arsenic present in the soil along with consistent, but minor, contributions from arsenite. This confirms the expectation that As(III) added to the soil is, to a large extent, oxidized to As(V) over the 5 wk course of the growth experiment, and that this form is taken up by the ferns. This oxidation is probably slow and hence plants in the two different dosing regimens were likely to be exposed to a different profile of As chemical forms. We noted that an additional component, beyond As(V) or As(III) forms, was required to generate satisfactory fits to these four spectra; if only As(V) and As(III) were used in the fits, a significant residual was found in the energy range between the white line peaks of these model compound spectra. To our knowledge the only known potentially relevant classes of As compounds with white line peaks in this energy range are the thioarsenates^58^ and the methylarsenates (mono- and dimethyl arsenic acids). We found that the inclusion of the spectrum of solid sodium dithioarsenate significantly improved the XANES fits for these four spectra, including that of the *P. vittata* root region. Despite increased uncertainty in the XANES fits arising from suboptimal energy calibration and uncharacterized dithioarsenate model compound (see Experimental Methods - Synchrotron-based X-ray fluorescence and X-ray absorption near-edge structure imaging section), the fitting for these four spectra is consistent with previous literature examples.^58^ Pickering *et al*.^28^ showed that *P. vittata* root contained predominantly As(V) and that result is consistent with what we observe here. One must take into consideration that the root spectra likely contain a significant signal component from soil that is directly behind the root in these samples (a thin root has low absorption of As Kα X-ray emission). We suspect that the thioarsenate-like species is present in the soil rather than in the root, based on what other groups have observed previously, but further experiments would be required to demonstrate this unequivocally. Nevertheless, in *P. calomelanos*, in contrast to *P. vittata*, the dominant As species in roots is As(III) followed by As(V), and As(III) glutathione [As(GSH)_3_], which means that some of the As(III) is complexed with glutathione, or a related species capable of binding As through a thiolate, such as a phytochelatin, in the root. Spatially mapping these chemical species broadly as As(III) and As(V) components reveals that in the *P. calomelanos* roots As(III) dominates, while in *P. vittata* roots As(V) clearly dominates (Supplementary Fig. S6).

**Table 4.** Results of linear combination fitting of model compound spectra to As K-edge XANES spectra extracted from specified spatial regions of rhizoboxes XANES imaging data (refer to Fig. 8). Refer to Experimental Methods - Synchrotron-based X-ray fluorescence and X-ray absorption near-edge structure imaging section for details regarding spectral models

	Proportion of component fitted							
Sample	As(GSH)_3_	Arsenite pH 7	Arsenite pH 10.5	Dithio-arsenate	Arsenate pH 4.5	Arsenate pH 9	*N*_tot_	residual (×10^−3^)
*P. calomelanos* root area [As(V)-spiked soil]	0.16	0.43		0.27	0.18		1.04	3.7
*P. calomelanos* soil area [As(V)-spiked soil]		0.08		0.22		0.74	1.04	4.7
*P. calomelanos* root area [As(III)-spiked soil]	0.13	0.29	0.29	0.16		0.16	1.02	3.4
*P. calomelanos* soil area [As(III)-spiked soil]		0.12		0.20		0.72	1.04	4.4
*P. vittata* root area [As(III)-spiked soil]		0.20		0.29	0.12	0.42	1.03	2.6
*P. vittata* soil area [As(III)-spiked soil]		0.09		0.27	0.10	0.57	1.03	3.4

## Discussion

In this study, the use of rhizoboxes revealed the main similarities and differences in *P. calomelanos* and *P. vittata* roots growing in As(III) and As(V)-spiked soils. In both species As cycles throughout the root systems taking up As from the enriched side and transporting it to the roots growing in the control side soil (Figs. 5 and 6). This suggests that As is remobilized along with other macro- and micronutrients throughout their roots.^60,61^ Morphologically, both ferns grew healthy roots in control and As-rich soils, although *P. calomelanos* roots developed more root hairs than *P. vittata* (see Figs. 3 and 6). The significant difference was in the biomass and As accumulation in roots and fronds, while *P. calomelanos* outgrew, *P. vittata* was more efficient taking up As, by up to three-fold (Fig. 1, Table 3). This efficient translocation in *P. vittata* has been reported at up to four-fold compared to *Pteris quadriaurita*, an As hyperaccumulator.^29^ In *P. vittata*, the As translocation efficiency is regulated by the rhizome, which can switch the transport of As from young fronds to mature ones under high As exposure.^31,32,62^ The As speciation in this species is also regulated with aging processes, As(III) predominates in younger fronds, while As(V) in older ones.^63^ Furthermore, *P. vittata* rhizodermis and root hairs were reported to be rich in pectin, and a lignified cortex, both associated with high accumulation capacity of ions.^64^

The As mobility and uptake by plants is determined by the soil biogeochemical processes.^65^ Although total concentration in the soil was set to 100 µg As g^−1^ in our study, the [Sr(NO_3_)_2_-extractable] analysis showed that only ∼4 µg As g^−1^ was phytoavailable in both As(III) and As(V) enriched soil, which means that As is adsorbed by iron and aluminum oxides/hydroxides, limiting the bioavailability.^25,66^ Arsenite mobility and solubility is higher than As(V), up to 25–60 times,^67^ in this study, the phytoavailable fraction in the As(III)-spiked soil was slightly higher (3.55–4.21 µg As g^−1^) than As(V)-spiked soil (2.61–4.06 µg As g^−1^). However, both ferns translocated slightly more As from the C | As(V) treatment compared to the C | As(III) treatment, and the highest accumulation for both ferns was recorded in the mixed treatment As(V) | As(III) (Fig. 1a), with 6670 µg As g^−1^ as average in old fronds of *P. vittata* and 4020 µg As g^−1^ in young fronds of *P. calomelanos*. Similar to our results, *P. vittata* did not show significant difference in As uptake when subjected to As(V) and As(III) treatments in a concentration of 10 mg As L^−1^.^68^ Even though As(V) predominates under aerobic conditions, As(III) can also be present due to microorganism activity and root exudates,^69,70^ coexisting both As species in the substrate.^71^

We found a predominance of As(V) in soils, even in As(III)-dosed soil. In agreement with our findings, arsenite in soil can be oxidized to As(V), especially in presence of Fe-hydroxides, Al and Mn^72^; e.g. the As(III) recovery after the spike of 4 µg As(III) g^−1^ was 1.1 µg As(III) g^−1^ in soils with higher content of Fe, Al and Mn, in comparison to a similar treatment but with lower Fe, Al, and Mn in soils, where the As(III) recovery was 2.55 µg As(III) g^−1^.^73^ Moreover, the rate of As(III) oxidation in spiked soils depends on the biogeochemical characteristics of the soil, e.g. if Fe-oxyhydroxides are present in the soil, they can adsorb As(III) on the surface, stabilizing As(III) and coexisting with As(V).^66,74,75^ Likewise, microbial activity added to the influence of oxides lead to partial or total As(III) oxidation in spiked soils, limiting the measurement of this species by XANES method.^75^ Also, chemical reactions during the extraction method to identify the proportion of As(III) and As(V) in soils can yield misleading data. The high intensity of X-rays used in XANES analysis can lead to As(V) reduction to As(III) in As(V)-spiked soils.^38^ The stability of As(III) over time, especially in spiked soils, has not been concluded yet, and may be susceptible to slow oxidation.^75^ Furthermore, photodamage might occur, during the XANES analysis, but our previous work showed that this was very limited in XANES imaging mode compared to confocal spot mode.

The oxidation reduction potential in the soil for this experiment was 224 ± 2.52 mV.^47^ The exchangeable sulfur (S) range was 32.1–117, mean: 58.8 µg S g^−1^,^76^ and the pH was 5.28 in the As(III)-spiked soil (Table 1), providing a moderately reducing environment, where the formation of thioarsenates can be possible,^26^ given high S available,^77^ as it was identified in this study by XANES (Table 4). In strongly reducing environments, the high affinity between As(III) and S permits the oxidation of As(III) to As(V) in the form of thioarsenates.^78^ In laboratory studies, the dissolution of orpiment (As_2_S_3_) in neutral to alkaline pH produces thioarsenates of 43–55% of total As.^79^ Microbial production of sulfur can enhance the production of thioarsenates,^80^ and conversely sulfur-oxidizing bacteria can transform it into As(V).^81^ Thioarsenates have a closer edge position to As(III) than As(V), and may be mistakenly identified as As(III) with XANES in mixed As-S compounds.^58^ Furthermore, thioarsenates are not stable and are sensitive to oxidation and pH and are converted to As(V) and As(III).^78,82,83^ Thioarsenate adsorption capacity is weaker than As(III) and As(V), making As more mobile^84,85^; thioarsenates are more toxic to plants than As(V).^26^

In our study we found that *P. vittata* roots are mainly enriched with As(V), similar to previous reports,^28^ although this result differs substantially from a previous investigation which reported preferential As(III) storage in *P. vittata* roots.^29^ In a previous study, As(III)-oxidizing bacteria in the rhizosphere of *P. vittata* were suggested to contribute to the As cycling, oxidizing the As(III) efflux from the roots to As(V).^86^*Pityrogramma calomelanos* roots differ from those of *P. vittata*, storing mainly As(III) followed by As(V) and As-glutathione [As(GSH)_3_]. The complexation of As with glutathione is a mechanism that plants develop to cope with the As toxicity.^87^*P. calomelanos* accumulates As less efficiently than *P. vittata*, which depletes more As even from the control soil (1.83 µg g^−1^ for *P. vittata* and 2.79 µg g^−1^ for *P. calomelanos*) and As(V)-spiked soil (85.1 µg g^−1^ and 92.8 µg g^−1^, respectively) (Table 2). *P. vittata* roots accumulate approximately three-fold As in roots in comparison to *P. calomelanos* across the different treatments, e.g. in the As(V) | As(III) treatment *P. vittata* has 762 µg As g^−1^ in roots growing in the As(III) section, and *P. calomelanos* 131 µg As g^−1^ in roots in the same section. Higher As concentration was reported in *P. vittata* compared to *P. calomelanos*, albeit the translocation to the aerial tissues essentially correlated to the type of soil. In kurosol and vertosol soils translocation by *P. vittata* was stronger, and in ferrosol soils translocation by *P. calomelanos* was stronger, showing that As translocation in both ferns depends on soil properties, i.e. free Fe, clay and organic matter content.^88^ Contradicting Xu *et al*.’s results, *P. calomelanos* was reported to attain higher As accumulation in fronds (887 µg As g^−1^) compared to *P. vittata (*674 µg As g^−1^) after growing for 10 mo in a former cattle dip area, polluted with As (830 µg g^−1^ on average) in New South Wales, Australia.^89,90^

The As distribution in the roots of *P. calomelanos* and *P. vittata* in the As-enriched soil displays a higher As concentration, compared to the control soil, where As cycles in lower quantity, although with some cumulus of high As in the joint of primary and lateral roots (Figs. 5–7). It has been reported that *P. calomelanos* darkened and shortened lateral roots as a response to As enriched nutrient solution (30 mM As ∼ 2250 µg As g^−1^).^34^ In our study, conversely, lateral roots, root hairs and root tips grew healthy across all the treatments, and only primary roots were darkened. However, in some plant species lateral roots and root hairs are developed when P bioavailability is low in the substrate, as a response to nutrient deficiency, triggered by hormonal signals, i.e. auxin hormone.^91–93^ The emergence of lateral roots from inner tissues (pericycle or endodermis) implies a series of challenging molecular and cellular regulatory processes.^94–96^ We postulate that As accumulation in nodes of the joints of lateral and primary roots is a mechanism to regulate the amount of As in younger lateral roots. It is clearly visible in *P. vittata*, that the As is transported through the vascular bundle and enriched in the base of lateral roots (Fig. 7).

Most plants develop different strategies to avoid toxic elements in the substrate, i.e. secreting organic acids to restrict the influx of metals by chelation^97^ or changing the root structure to inhibit root growth in areas where toxic elements are present.^98,99^ In the case of As, as it is not essential for plants, As(V) is taken up inadvertently through phosphate transporters in aerobic environments, and As(III) by silicon transporters in anaerobic media; then sulfur through complexation of arsenite by thiol-rich peptides retains As in the roots.^25,28,100^ In our study, both ferns translocate As from roots to fronds efficiently, in fronds the As enrichment is in the rachis (mid-rib of the blade), costa (midrib of the pinna), and higher in the terminal veins in old specimens, which is consistent with previous studies.^27,39^ In *P. vittata*, As is stored in the endodermis and pericycle of the rachis and pinnules.^19^ Arsenic hyperaccumulators predominantly store As(III), despite As(III) being more toxic than As(V).^101^ Both As species are detrimental for plants and both affect plant metabolism; however, As(V) can compete with phosphate and interfere in cellular energy production disrupting ATP processes, while arsenite is a dithiol reactive compound and can inactivate enzymes and proteins inhibiting cellular function.^102^

In this study, we focused on the speciation of arsenic in soil and roots of the two best arsenic hyperaccumulators *P. vittata* and *P. calomelanos*. However, the rhizosphere is a complex environment in which microbes (rhizobiome) play an essential role in As speciation, mobility, and plant growth.^103–105^ More scientific reports on the microbiome were published for *P. vittata* in comparison to *P. calomelanos*. For example, *P. vittata* produces more exudates than normal plants, allowing the development of a microbiome responsible for the mobilization of As in the soil through As reduction, oxidation, methylation, and demethylation, and As sorption and desorption.^106,107^ Bacteria isolated from *P. vittata* roots can solubilize As from insoluble ferric arsenate (FeAsO_4_) and aluminum arsenate (AlAsO_4_) minerals and enhance As uptake.^108^ A study collected 864 bacterial cultures from the rhizosphere of *P. vittata*, and the majority tolerated As(V) more than As(III), 95% promoted As (V) reduction, 73% As(III) oxidation, and 71% reduced and oxidized As.^109^ Another study reported a diverse As(III)-resistant bacteria in *P. vittata* roots growing in soils with up to 24.5 µg As g^−1^.^110^ We suggest further studies (i.e. metagenomics, transcriptomics) to better understand the interactions of the root exudates and microbiome in the soil and As hyperaccumulator ferns, especially for *P. calomelanos*.

## Conclusions

This study has shown that *P. calomelanos* and *P. vittata* grow vigorously in nutrient-poor and As-rich soils, cycling the As along the roots, and the As chemical species accumulated in their roots is differentiated. Further research is required to explore the full potential of both ferns for As phytoextraction, since *P. vittata* is highly effective, but *P. calomelanos* develops more biomass, an important characteristic for successful phytoextraction.^8,16^ During the last 10 yr, above ground tissues of hyperaccumulator plants have been extensively studied by novel methods to reveal their mechanisms to store metals. However, roots are still the hidden puzzle of the hyperaccumulation phenomena, and even less is known about the rhizosphere interface and the processes that lead to metal(loid) uptake by hyperaccumulator plants. In this study, we have compared root preferences, and active As-uptake in the strongest As hyperaccumulator ferns. The dominant form of As in soils was As(V), even in As(III)-dosed soils, and the major form in *P. calomelanos* roots was As(III), while it was As(V) in *P. vittata* roots. Arsenic was cycled from roots growing in As-rich soil to roots growing in control soil.

## Supplementary Material

mfad047_Supplemental_File

## Data Availability

The data underlying this article will be shared on reasonable request to the corresponding author.
