# Supplementary material for: Chemical transformations of arsenic in the rhizosphere–root interface of Pityrogramma calomelanos and Pteris vittata
Source: Metallomics. 2023 Aug 1;15(8):mfad047. doi: 10.1093/mtomcs/mfad047 (PMC10427965; doi:10.1093/mtomcs/mfad047)
Supplement: mfad047_Supplemental_File [file mfad047_supplemental_file.pdf]

SUPPLEMENTARY INFORMATION

Chemical transformations of arsenic in the rhizosphere-root interface of *Pityrogramma calomelanos* and *Pteris vittata*

Amelia Corzo Remigio[1], Hugh H. Harris[2], David J. Paterson[3],

Mansour Edraki[1], Antony van der Ent[1,4,5*]

[1]Centre for Mined Land Rehabilitation, Sustainable Minerals Institute, The University of Queensland, Australia.

[2]Department of Chemistry, The University of Adelaide, Australia.

[3]Australian Synchrotron (ANSTO), Clayton, Victoria 3168, Australia.

[4]Laboratory of Genetics, Wageningen University and Research, The Netherlands.

[5]Laboratoire Sols et Environnement, INRAE, Université de Lorraine, France.

*Corresponding author: (antony.vanderent@wur.nl)

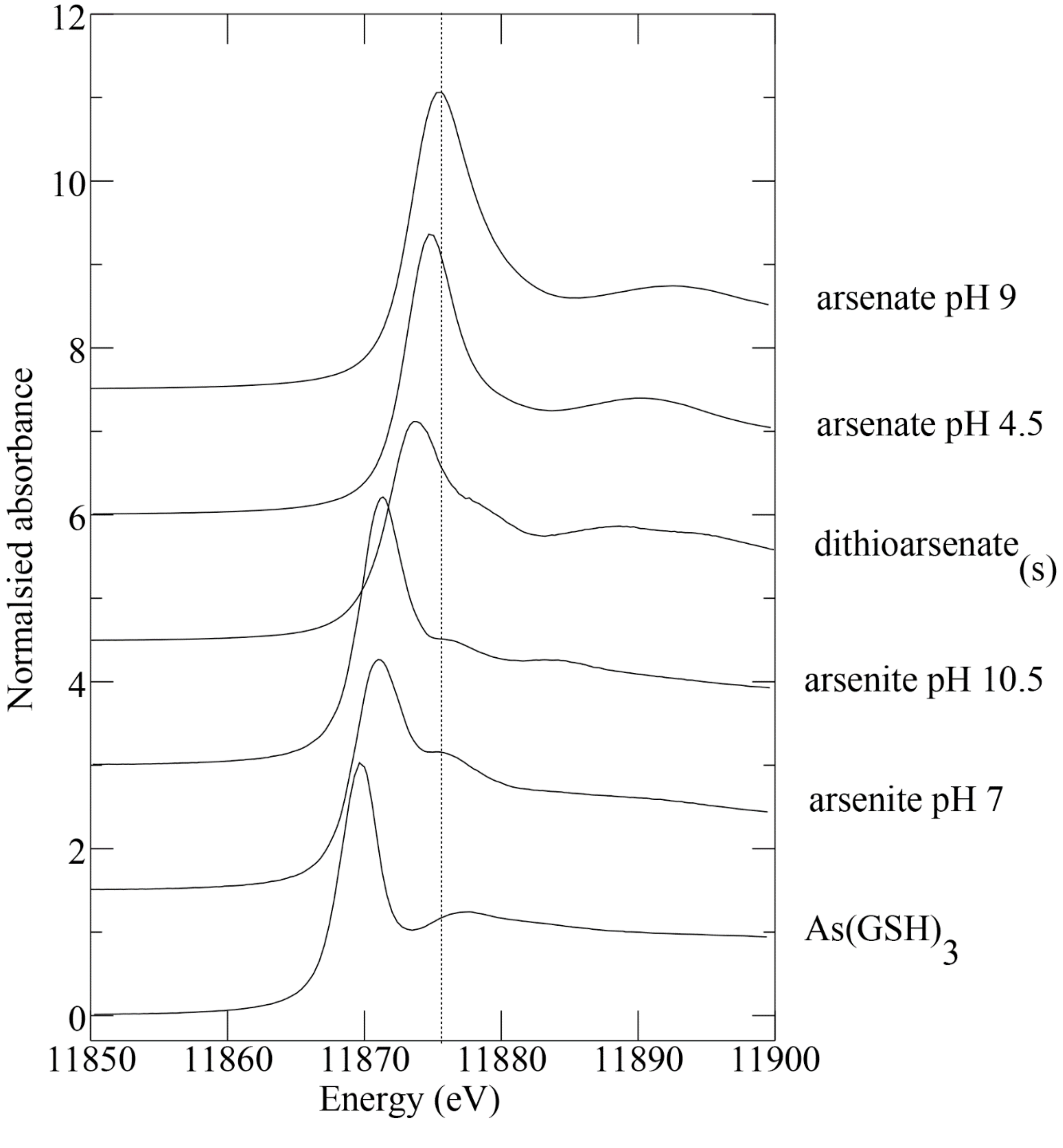

Supplementary Figure 1. As K-edge X-ray absorption near edge spectra of the six selected model compounds. The dashed vertical line indicates 11875.5 eV.

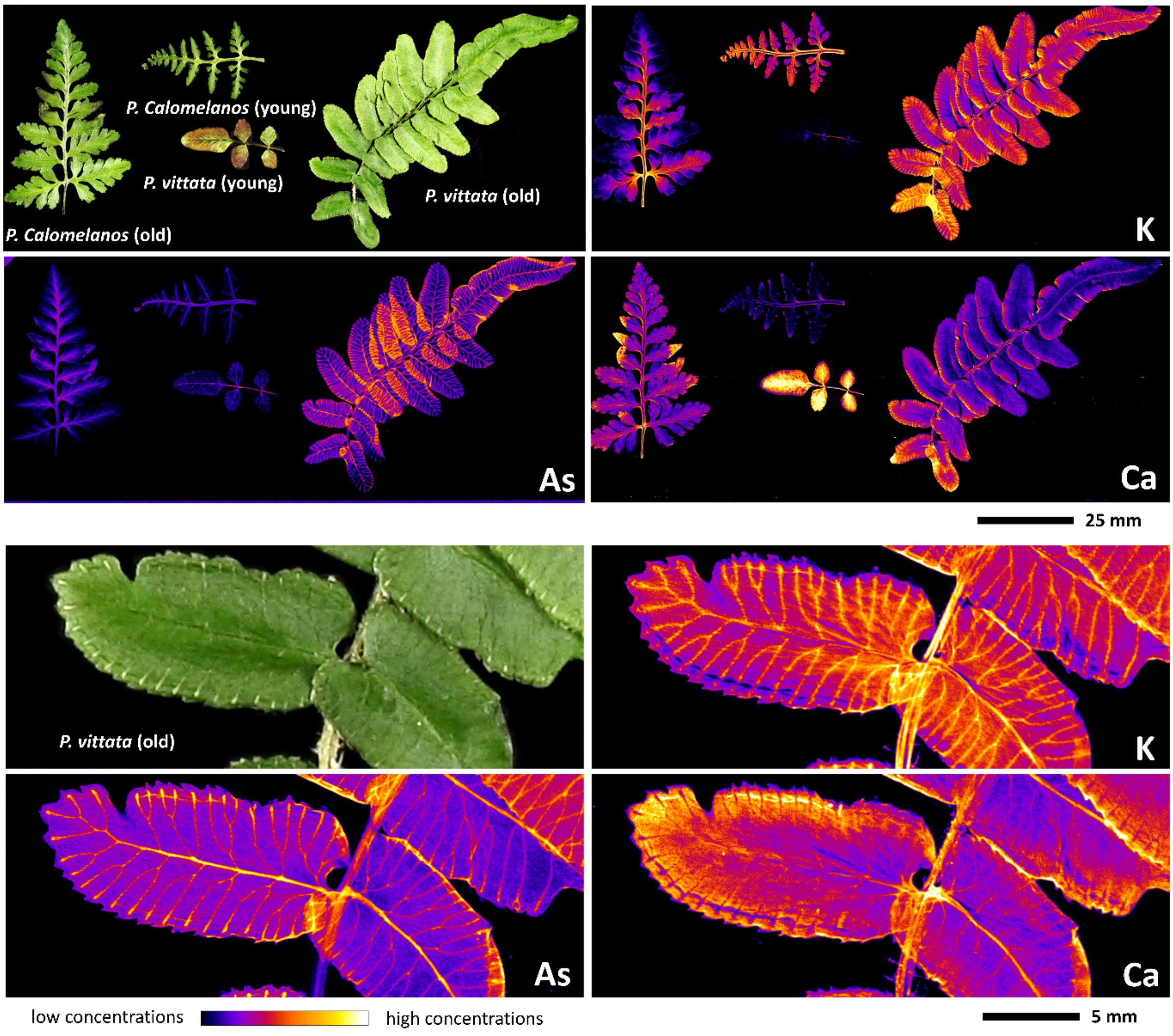

Supplementary Figure 2. µXRF map distribution of K, As and Ca of *Pityrogramma calomelanos* and *Pteris vittata* both collected from the treatment C | As(III) (100 µg g^{-1}) replicate 1. On top younger and older blades of both fern species, on bottom a magnified map of older blades of *Pteris vittata*.

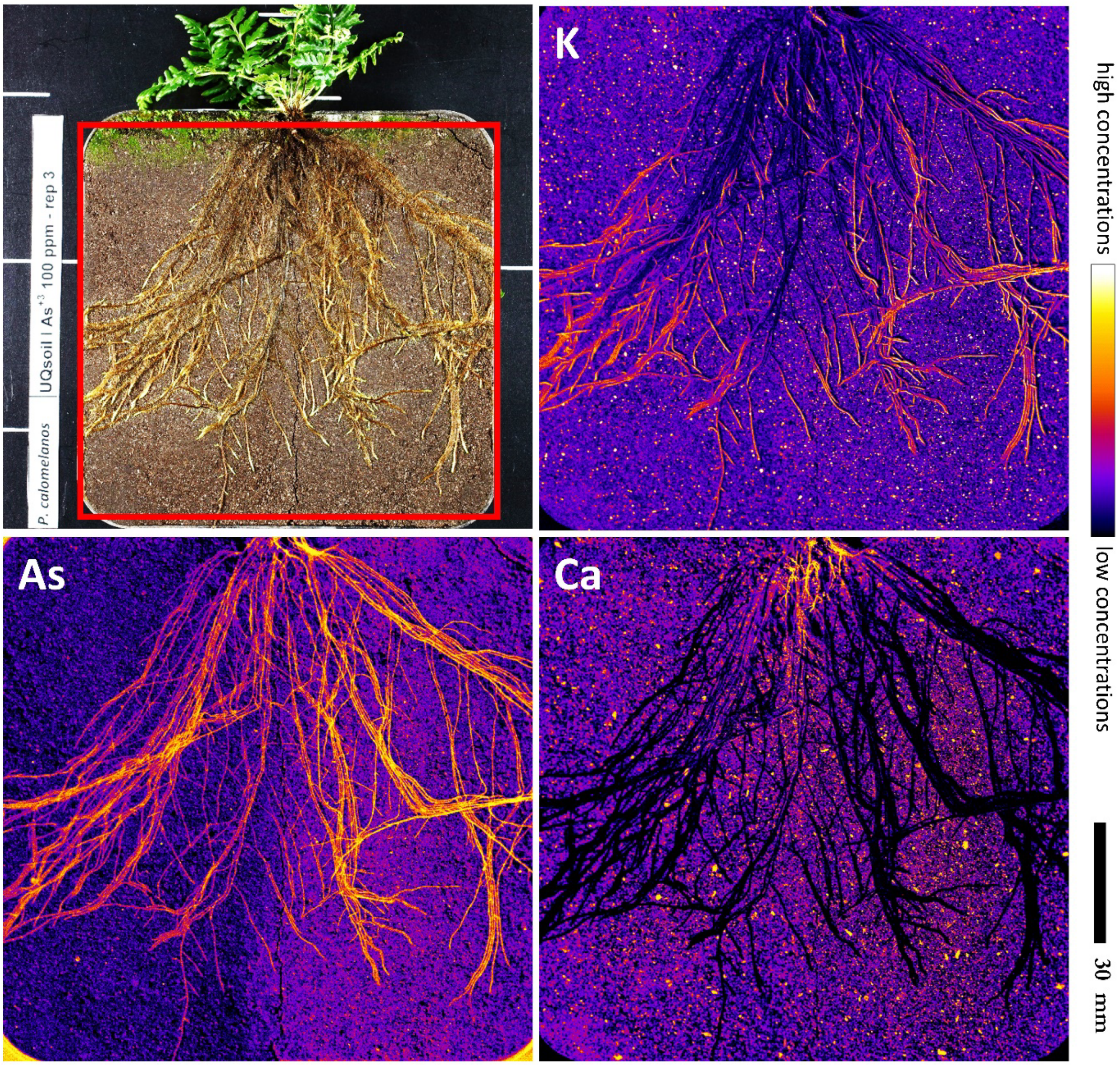

Supplementary Figure 3. µXRF map distribution of K, As and Ca of the roots of *Pityrogramma calomelanos* growing in the treatment C ǀ As(III) (100 µg g^{-1}) replicate 3.

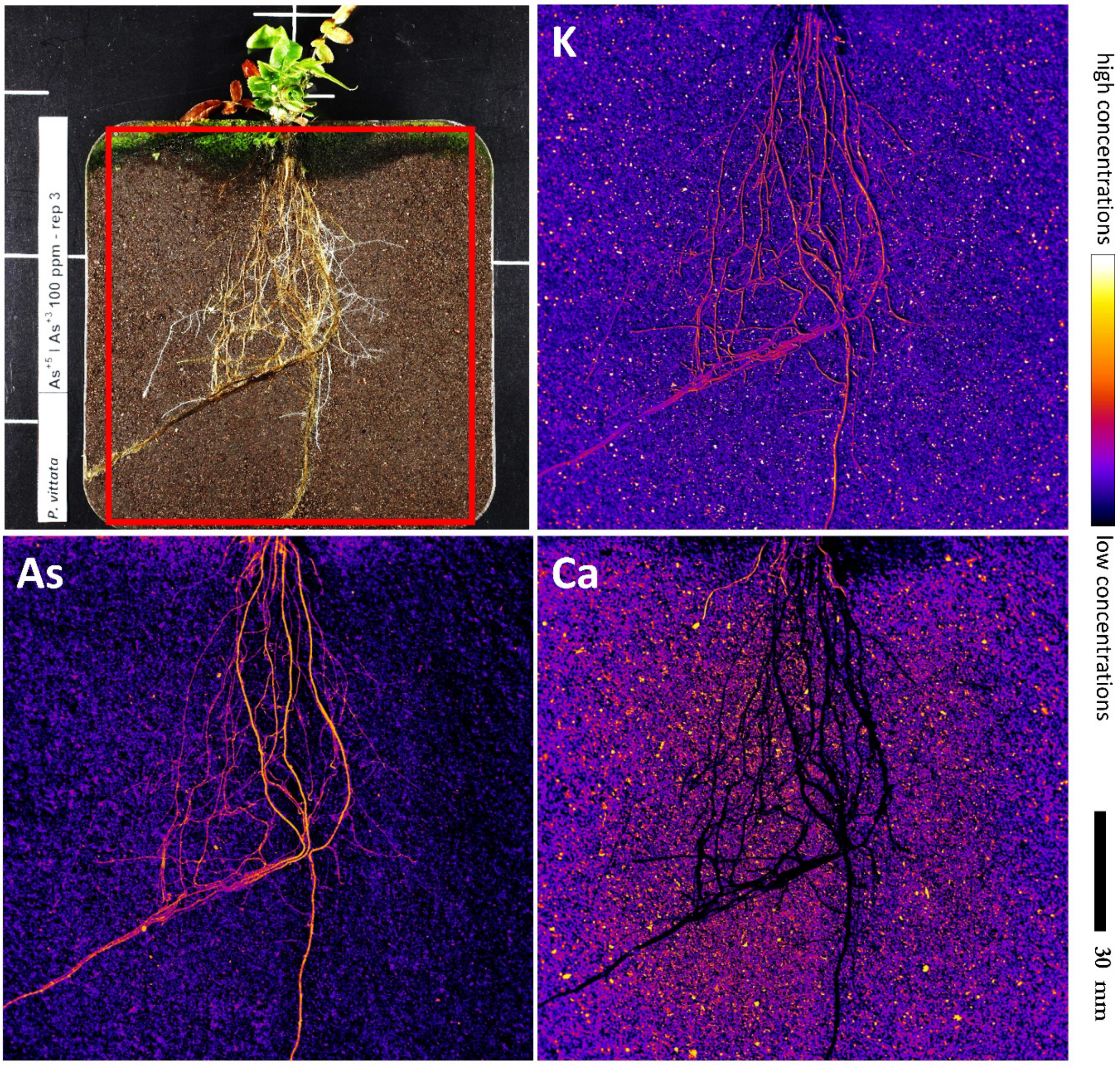

Supplementary Figure 4. µXRF map distribution of K, As and Ca of the roots of *Pteris vittata* growing in the treatment As(V) | As(III) (100 µg g^{-1}) replicate 3.

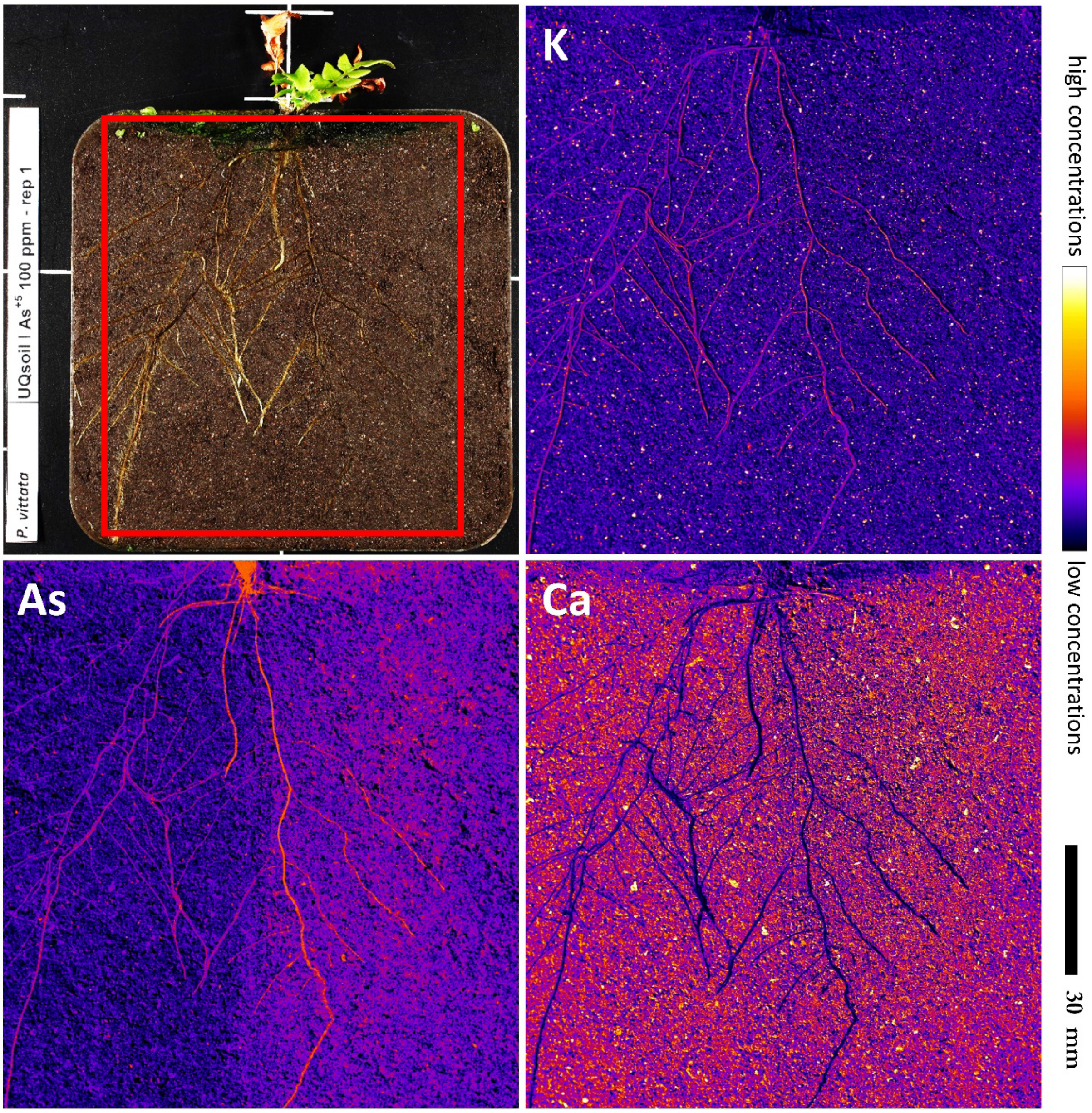

Supplementary Figure 5. µXRF map distribution of K, As and Ca of the roots of *Pteris vittata* growing in the treatment C | As(V) (100 µg g^{-1}) replicate 1.

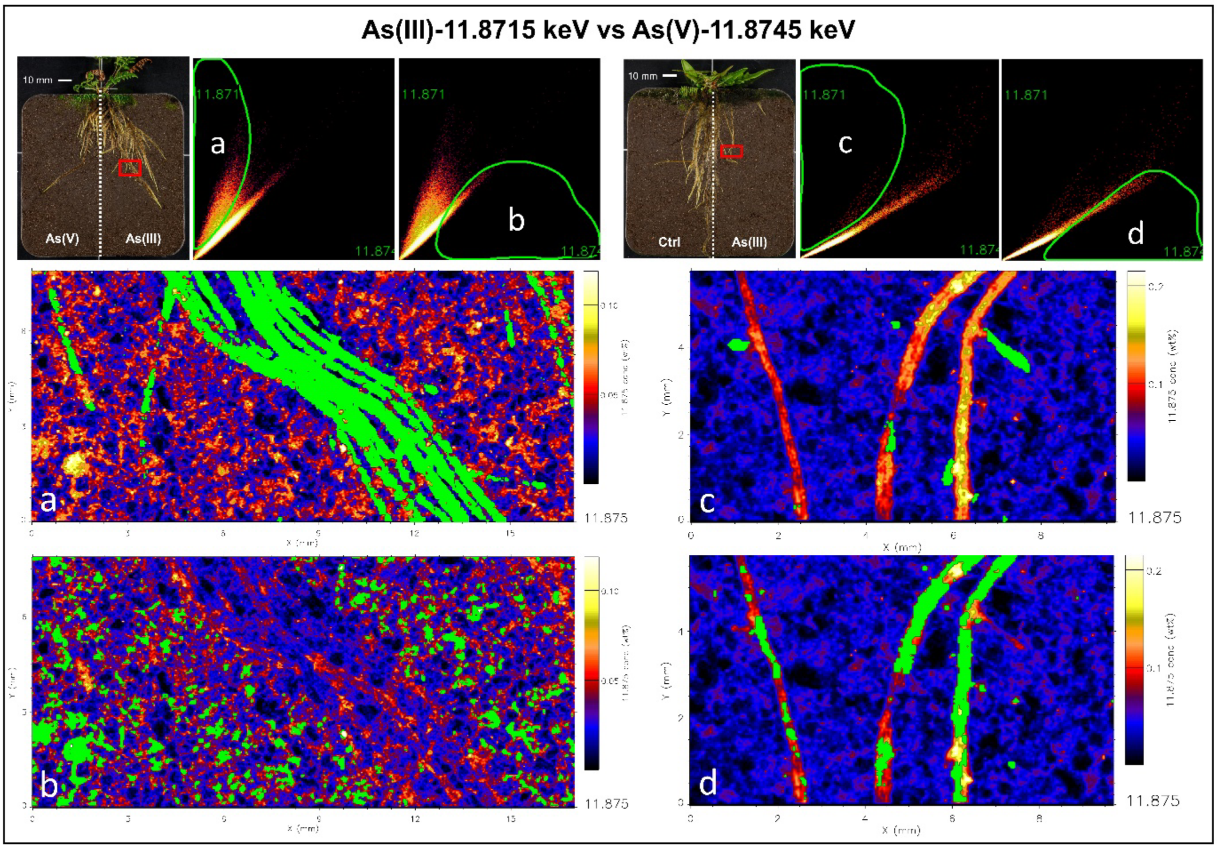

Supplementary Figure 6. Association energies between As(III) vs As(V) for *P. calomelanos* in As(III)-enriched soil (left section), and *P. vittata* As(III)-enriched soil (right section). The scatter plots on the top row display data points for each spatial pixel in the XANES images, plotted to show the relative signal intensity of that image pixel at the two incident X-ray energies shown on each of the x- (11.871 keV) and y-axes (11.874 keV). These two energies correspond to the peak absorbance energies in the XANES spectra of As(III) and As(V), respectively. On top, the letters represent the regions selected to observe the predominance of that particular As species in the distribution map. On the bottom, these pixels are marked in green in the maps: (a) As(III) species in *P. calomelanos* dominate in the roots; (b) As(V) species occur across the soil; (c) As(III) species in *P. vittata roots* are low; (d) As(V) species occur mainly in *P. vittata* roots.
